# Comparative Analysis of Gut Microbiota Among the Male, Female and Pregnant Giant Pandas (*Ailuropoda Melanoleuca*)

**DOI:** 10.1515/biol-2019-0032

**Published:** 2019-07-23

**Authors:** Siyue Zhao, Caiwu Li, Guo Li, Shengzhi Yang, Yingming Zhou, Yongguo He, Daifu Wu, Yu Zhou, Wen Zeng, Ti Li, Yuanyuan Qu, Bei Li, Wenwen Deng, Lei Jin, Xiumei Yu, Yan Huang, Hemin Zhang, Likou Zou

**Affiliations:** 1 Department of Applied Microbiology, College of Resources, Sichuan Agricultural University, 611130, Chengdu, Sichuan, China; 2 China Conservation and Research Center for Giant Panda, 611830, Dujiangyan, Sichuan, China; 3 Key Laboratory of State Forestry and Grassland Administration on Conservation Biology of Rare Animals in The Giant Panda National Park (China Conservation and Research Center of Giant Panda), 611830, Wolong, China

**Keywords:** giant panda, gut microbiota, high-throughput sequencing, gender, pregnant

## Abstract

The giant panda (GP) was the most endangered species in China, and gut microbiota plays a vital role in host health. To determine the differences of the gut microbiota among the male, female and pregnant GPs, a comparative analysis of gut microbiota in GPs was carried out by 16S rRNA and ITS high-throughput sequencing. In 16S rRNA sequencing, 435 OTUs, 17 phyla and 182 genera were totally detected. *Firmicutes* (53.6%) was the predominant phylum followed by *Proteobacteria* (37.8%) and *Fusobacteria* (7.1%). *Escherichia/Shigella* (35.9%) was the most prevalent genus followed by *Streptococcus* (25.9%) and *Clostridium* (11.1%). In ITS sequencing, 920 OTUs, 6 phyla and 322 genera were also detected. *Ascomycota* (71.3%) was the predominant phylum followed by *Basidiomycota* (28.4%) and *Zygomycota* (0.15%). *Purpureocillium* (4.4%) was the most prevalent genus followed by *Cladosporium* (2.5%) and *Pezicula* (2.4%). Comparative analysis indicated that the male GPs harbor a higher abundance of phylum *Firmicutes* than female GPs with the contribution from genus *Streptococcus*. Meanwhile, the female GPs harbor a higher abundance of phylum *Proteobacteria* than male GPs with the contribution from genus *Escherichia/ Shigella*. In addition, the shift in bacteria from female to pregnant GPs indicated that phylum *Firmicutes* increased significantly with the contribution from *Clostridium* in the gut, which may provide an opportunity to study possible associations with low reproduction of the GPs.

## 1 Introduction

In 2013, nearly 1,860 individual giant pandas (GP, *Ailuropoda melanoleuca*) were found in Western China (http://www.forestry.gov.cn/main/72/content-742880.html). The GP is a rare wild animal, ranking at the top of the list of endangered species on earth [1]. It is well known that the GP harbors a special dietary preference to bamboo, a high-fiber food. Although GP belongs to the Order *Carnivore* [2], it consumes a unique herbivorous diet [3]. Previous studies showed that about 8% and 27% of the cellulose and the hemicelluloses, respectively, in bamboo could be digested by GPs [2]. Through whole-genome sequencing, however, no specific genes that are responsible for the digestion of cellulose and hemicellulose were found in GPs [4], suggesting that gut microbiota play a vital role in digesting bamboo fibers [2]. Besides, the gut microbiota also has an impact on the health of the host [5]. The gut microbiota is involved in energy harvesting and storage, as well as in a variety of metabolic functions such as fermenting and absorbing undigested carbohydrates [6]. More importantly, it has become clear that the gut microbiome plays a critical role in health, nutrition and physiology of wildlife, including numerous endangered animals in the wild and in captivity [7]. Disturbances to this community can have adverse impacts on animal health [8], which would not benefit the survival of wild animals.

The 16S ribosomal RNA (16S rRNA) and Internal Transcribed Spacer (ITS) high-throughput sequencing overcame the limitations of culture-based bacterial and fungal detection [9], and allowed exploration of the gut microbiota in depth, exhibiting its complete bacterial and fungal diversity [10,11]. In recent years, 16S rRNA and ITS high-throughput sequencing were also applied to analyze the gut microbiota community composition of GPs [12]. Using such sequencing, comparisons of the gut microbiota in GPs have been subsequently conducted, including the differences in age [9] and season [13]. However, the composition of the gut microbiota is able to be affected by various factors such as intestinal environment, nutritional and non-nutritional dietary components, antibiotic use [14] and gender difference [15]. In addition, gut microbiota has also been reported to be modified during pregnancy [16]. It has been reported that the GP has a low fecundity, which may be one of the causes behind its population decline [17]. According to previous studies, pregnancy has impact on the diversity of gut microbiota to some extent and the maternal intestinal microbiota is modified over the course of healthy pregnancy. It is possible that maternal gut bacterial profiles may be associated with the known endocrine changes that accompany the female reproductive (estrous) cycle [18]. On the other hand, the microbiota could lead to host maternal gestational weight gain after pregnancy [19]. In addition, some bacteria could cause host adverse pregnancy outcomes [20]. Therefore, there may be a strong relationship between gut microbiota and pregnancy. The variation of gut microbiota should also have an effect on pregnancy. Hence the variation or difference in gut microbiota during pregnancy should also be examined to test this hypothesis.

Until now, the differences of gut microbiota composition among male, female and pregnant GPs have not been examined. Here, the 16S rRNA and ITS high-throughput sequencing were used to characterize the gut microbiota among male, female and pregnant GPs in order to make a comparative analysis of gut bacterial and fungal communities.

## 2 Materials and Methods

### 2.1 Sample collection

This study was carried out with approval of the China Conservation and Research Center for Giant Panda in Sichuan, China. All the giant pandas were fed the same diet. Eighteen fecal samples were collected from the adult male (n=7), female (n=5) and pregnant (n=6) GPs (6 to 10 years old) once in 2016. The GPs had similar husbandry conditions and were housed at Bifengxia Base, China Conservation and Research Center for Giant Panda. Fecal samples were collected immediately after defecation, snap frozen (-80ᵒ C), and shipped to the laboratory in dry ice.

### 2.2 DNA extraction and Miseq sequencing

The whole genome DNA from the samples was extracted by using the Mobio Power Fecal^TM^ DNA Kit (Laboratories Inc., America) and EZNA Fungal DNA Mini prep Kit (Omega Inc., America) according to the manufacturer’s instruction. The hypervariable region V4 of the 16S rRNA genes was amplified by PCR, using primers 520F (5’- barcode + AYTGGGYDTAAAGNG-3) and 802R (5’-TACNVGGGTATCTAATCC-3’); the first region of the ITS genes were amplified by PCR, using primers ITS1 (5’-barcode + TCCGTAGGTGAACCTGCGG-3’) and ITS2 (5’-GCTGCGTTCTTCATCGATGC-3’). The PCR products were then submitted to the Shanghai Personal bio-tech Co. Ltd for sequencing that based on an Illumina MiSeq 2500 platform [21].

### 2.3 Data analysis

Paired-end reads were assembled using FLASH (V1.2.7, http://ccb.jhu.edu/software/FLASH/) [22], after removing the barcode and primer sequence. High-quality clean tags were obtained according to the QIIME (V1.7.0, http://qiime.org/index.html) [23] quality control process. According to the reference database (Gold database, http://drive5.com/chime/uchime_download.html), the chimera sequences were detected using UCHIME algorithm (Algorithm, http://www.drive5.com/usearch/manual/uchime_algo.html) [24]. After chimera removal, the Effective Tags were finally obtained. Sequence analyses were performed using the Uparse software (Uparse V7.0.1001) [21]. Sequences with ≥ 97% similarity were assigned to the same Operational taxonomic units (OTUs). A representative sequence for each OTU was screened for further annotation. Based on OTUs and species annotation, the dominant species in various samples (groups) and OTU differential abundance testing information were determined.

Six indices including observed species, Chao1, Shannon, Simpson, ACE and Good’s coverage were used to analyze the complexity of species diversity for all samples. All indexes (including observed species, Chao1, Shannon, Simpson, ACE and Good’s coverage) for bacteria and fungi were analyzed by using software R (https://www.r-project.org/) with ANOVA (Analysis of Variance) method. Rarefaction curves and Rank abundance curves were delineated to evaluate the reasonableness of all the samples.

A one-way analysis of similarity (ANOSIM) [25] was performed to determine the differences among the male, female and pregnant groups. The principal coordinate analysis (PCoA) that using the OTU-based weighted Unifrac distance matrix was performed basing on “Out table” by using software R (https://www.r-project.org/) with package GUniFrac, ape and ggplot2 [26] to visualize the discrepancy among the male, female and pregnant groups. Meanwhile, a Venn diagram was employed to describe the common and unique OTUs in each group. The top 10 phyla and 35 genera were chosen to generate the percentage-stacked histogram of relative abundance for each sample and group respectively. According to the PCoA, the specific species that had significant difference among the 3 groups at each level were calculated by using T test and LDA effect size (LEfSe) analysis.

All raw sequences obtained in this study were archived at NCBI Sequence Read Archive (SRA) under accession number SRP149033.

## 3 Results

### 3.1 Overview of the sequencing data

In 16S rRNA sequencing, 1,148,722 high quality reads were obtained, and classified into 435 OTUs with the 97% similarity from the 18 fecal samples of the GPs. In ITS sequencing, 1,052,318 high quality reads were obtained, and classified into 920 OTUs with the 97% similarity.

The Alpha diversity indices for bacteria and fungi (including observed species, Shannon, Chao1, ACE and Good coverage) were shown in Table S1 and Table S2, respectively. However, there is no significance in all alpha diversity indexes (including observed species, Shannon, Chao1, ACE and Good coverage) among the male, female and pregnant groups (*p*>0.05). The rarefaction curves became flat gradually and reached a plateau with more data indicating that the number of OTUs for each sample was sufficient and reasonable (Fig.S1). The rank abundance curves that reflected the evenness and abundance of species in fecal samples horizontally and vertically, respectively, were demonstrated in Fig.S2.

### 3.2 Microbiota composition and relative abundance of all samples

**Figure 1:**
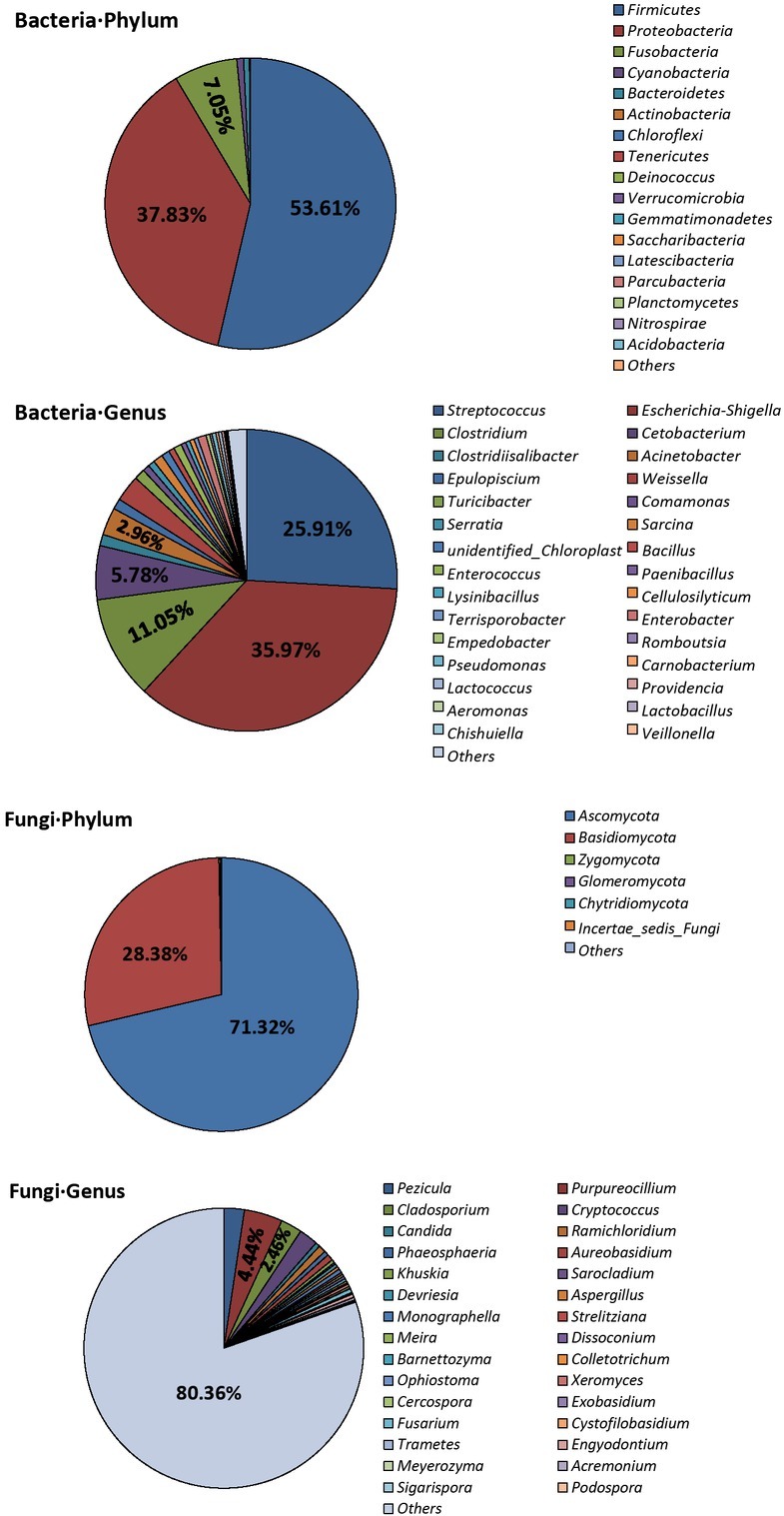
The relative abundance of bacteria and fungi at phylum and genus level in all samples.

For bacteria, we detected 17 phyla, 31 classes, 53 orders, 108 families and 182 genera in the gut microbiota community from these fecal samples of the GP (Fig. S3). *Firmicutes* (53.6%) was the predominant phylum followed by *Proteobacteria* (37.8%), *Fusobacteria* (7.1%), *Cyanobacteria* (0.74%) and *Bacteroidetes* (0.65%). *Escherichia/Shigella* (35.9%) was the most prevalent genus followed by *Streptococcus* (25.9%), *Clostridium* (11.1%), *Cetobacterium* (5.8%), *Acinetobacter* (2.9%), *Weissella* (2.8%), *Turicibacter* (1.2%), *Clostridiisalibacter* (1.2%), *Epulopiscium* (1.1%) and *Sarcina* (1.0%) (Fig.1).

For fungi, we detected 6 phyla, 32 classes, 99 orders, 189 families and 322 genera in the gut microbiota community from these fecal samples of the GP (Fig. S3). *Ascomycota* (71.3%) was the predominant phylum followed by *Basidiomycota* (28.4%), *Zygomycota* (0.15%), *Glomeromycota* (0.12%), and *Chytridiomycota* (0.02%). *Purpureocillium* (4.4%) was the most prevalent genus followed by *Cladosporium* (2.5%), *Pezicula* (2.4%), *Cryptococcus* (2.2%), *Ramichloridium* (0.85%), *Aureobasidium* (0.74%), *Phaeosphaeria* (0.54%), *Candida* (0.53%), *Monographella* (0.47%) and *Fusarium* (0.47%) (Fig.1).

### 3.3 Analysis of discrepancies for male, female and pregnant groups

**Figure 2:**
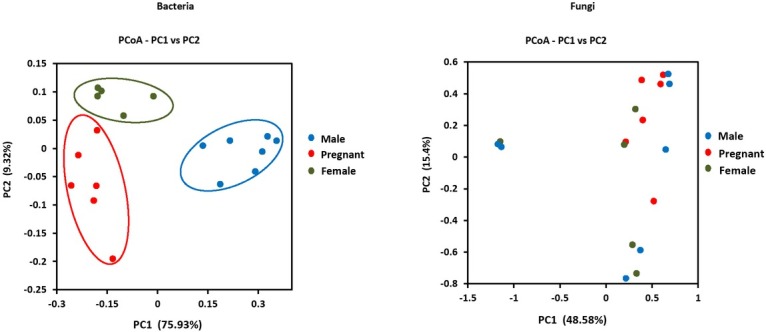
PCoA of the bacterial and fungal composition.

For bacteria, the principle coordinates analysis (PCoA) plots (Fig.2a) demonstrated that each group tended to assemble together within respective groups. Meanwhile, the PCoA result was verified by ANOSIM (R>0, P<0.01) (Fig.S4). For fungi, however, each group fails to assemble together within respective groups (Fig.2b). Therefore, the fungal samples were not used to make a group comparative analysis.

In Venn figures, 171 OTUs were found in all the groups shared by the male, female and pregnant group (Fig.S5). In addition, the shared OTUs comprised approximately 39.2% of the total OTUs, while 59, 30 and 52 OTUs were uniquely identified among the male, female and pregnant groups, respectively. Meanwhile, 9 phyla were found in all the groups shared by the male, female and pregnant groups (Fig.S5). Three phyla were uniquely identified in the female group. 76 genera were found in all the groups shared by the male, female and pregnant groups (Fig.S5). Moreover, 29, 12 and 19 genera were uniquely identified in the male, female and pregnant groups, respectively.

**Figure 3:**
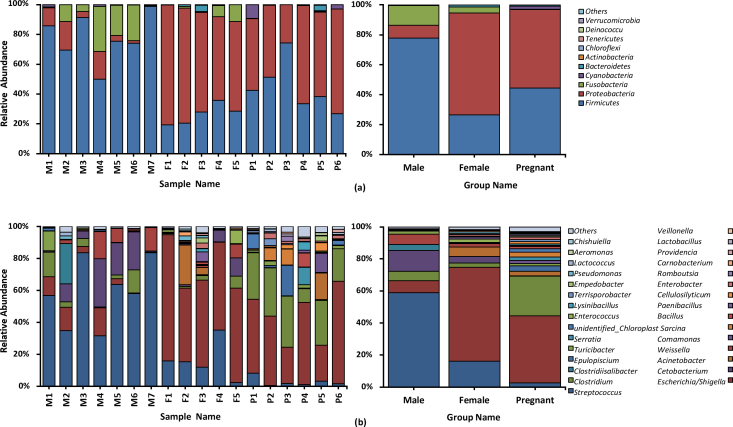
The composition of phyla (a) and genera (b) among the male, female and pregnant groups. Each bar represents the top ten bacterial species ranked by the relative abundance in each individual sample or group.

In order to exhibit the bacterial communities intuitively, we chose the top 10 phyla and 30 genera for each sample and group to generate the percentage stacked histogram of relative abundance (Fig.3). In the group of male GPs, *Firmicutes* (77.8%) was the predominant phylum followed by *Fusobacteria* (13.1%), *Proteobacteria* (8.6%), *Cyanobacteria* (0.38%) and *Bacteroidetes* (0.12%). *Streptococcus* (13.1%) was the most prevalent genus in male group which belonged to *Firmicutes* followed by *Cetobacterium* (13.1%), *Escherichia/Shigella* (7.4%), *Weissella* (6.4%), *Clostridium* (5.9%), *Clostridiisalibacter* (3.6%), *Turicibacter* (1.9%), unidentified *Chloroplast* (0.38%), *Pseudomonas* (0.35%) and *Lactococcus* (0.33%). It is noteworthy that *Clostridiisalibacter* is unique to the male GPs. In the female group, *Proteobacteria* (68.1%) was also the predominant phylum followed by *Firmicutes* (26.4%), *Fusobacteria* (4.1%), *Bacteroidetes* (1.2%) and *Actinobacteria* (0.11%). *Escherichia/Shigella* (58.7%) was the most prevalent genera followed by *Streptococcus* (16.2%), *Acinetobacter* (5.9%), *Cetobacterium* (4.1%), *Clostridium* (2.5%), *Enterococcus* (2.1%), *Weissella* (2.1%), *Paenibacillus* (1.3%), *Enterobacter* (0.98%) and *Pseudomonas* (0.89%). In the pregnant group, *Proteobacteria* (52.5%) was the predominant phylum followed by *Firmicutes* (44.4%), *Cyanobacteria* (2.2%), *Bacteroidetes* (0.68%) and *Fusobacteria* (0.17%). *Escherichia/Shigella* (41.8%) was the most prevalent genera followed by *Clostridium* (24.7%), *Epulopiscium* (3.4%), *Sarcina* (3.1%), *Acinetobacter* (2.9%), *Streptococcus* (2.7%), unidentified *Chloroplast* (2.2%), *Comamonas* (2.0%), *Serratia* (1.9%) and *Enterobacter* (1.7%).

No significance in all alpha diversity indexes (including observed species, Shannon, Chao1, ACE and Good coverage) was observed among the male, female and pregnant groups (*p*>0.05). The boxplot of Chao1 and Shannon index showed that diversity was not significantly different among the male, female and pregnant groups (Fig.S6).

**Figure 4:**
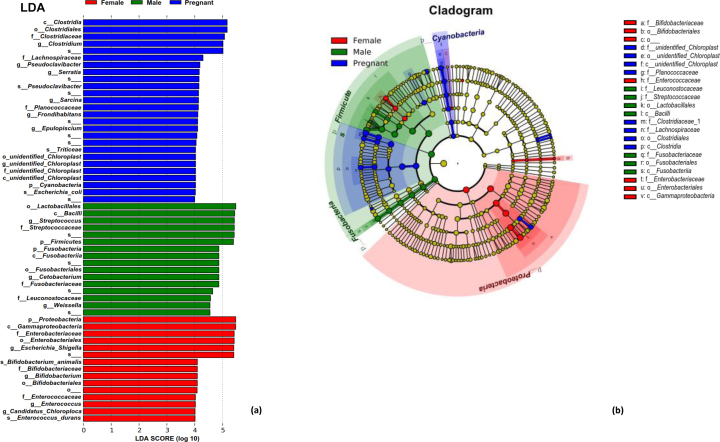
The results of LEfSe (LDA Effect Size) analysis (a). The histogram of LDA score showed the biomarkers with statistics difference among the groups. The influencing degree of species was expressed by the length of bar in histogram. In the cladogram (b), the circle radiated inside-out demonstrated the classification (from phylum to genus). Each small circle at different classification represented a taxa and the diameter of circle is proportional to the relative abundance. The species with no significance were colored by yellow and biomarkers were colored by different groups. Red and green dots represent the core bacterial populations in respective group.

**Figure 5:**
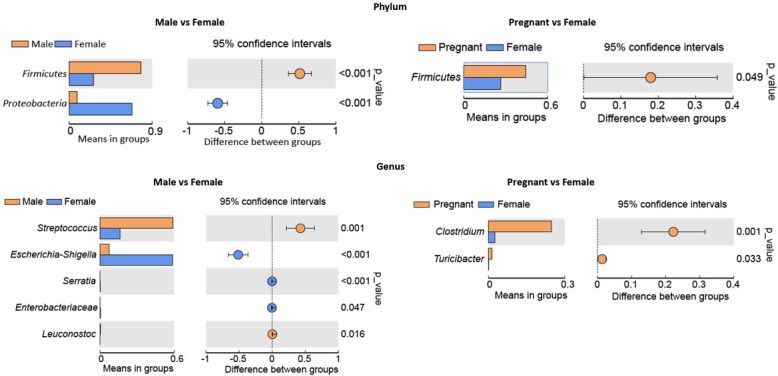
T-test bar plot, the species with significant discrepancy at phylum and genus level among the male, female and pregnant group, as well **Figure** as the **5**. relative abundance and p value.

The LDA effect size (LEfSe) analysis exhibits the specific taxa that had significant difference among male, female and pregnant groups (Fig.4a). A total of 16, 26 and 15 taxa that had discrepancies in relative abundance were presented in the male, female and pregnant groups, respectively (e.g. *Firmicutes*, *Proteobacteria*, *Streptococcus*, and *Escherichia/Shigella*). It’s obvious that, at the phylum level, the relative abundance of *Firmicutes* showed remarkable difference (*p*<0.001) in the male group, and *Proteobacteria* was significantly higher in the female group than other groups (*p*<0.001). The cladogram in Fig.4b showed the core bacterial species were in remarkable difference at all levels. According to the T-test of bacterial species difference, *Firmicutes* in the male group had a significantly higher abundance than female at the phylum level (*p*<0.001). The abundance of *Proteobacteria* in female group was significantly higher than that in the males (*p*<0.001). At the genus level, *Streptococcus* (*p*<0.001) and *Leuconostoc* (*p*<0.05) in male group showed a significantly higher abundance than female. The abundance of *Escherichia/Shigella* (*p*<0.001), *Serratia* (*p*<0.001) and *Enterobacteria* (*p*<0.05) were significantly higher in the female group than the male. According to the T-test of bacterial species difference, *Firmicutes* (*p*<0.05) in the pregnant group has a significant higher abundance than females at the phylum level. Meanwhile, the abundance of *Clostridium* (*p*<0.001) and *Turicibacteria* (*p*<0.05) in the pregnant group was also significantly higher than females at the genus level. More details on species with significant discrepancy at the phylum and genus level are presented in Fig.5.

## 4 Discussion

In this study, we characterized the gut microbiota among the male, female and pregnant GPs. Consistent with previous studies [3,13], all fecal samples had low diversity and were dominated by bacteria in the phyla *Firmicutes* (53.6%) and *Proteobacteria* (37.8%), with contributions from the genera *Escherichia/Shigella* (35.9%), *Streptococcus* (25.9%), and *Clostridium* (11.1%). Our study showed that most fungi in all fecal samples were affiliated to the phyla *Ascomycota* (71.3%) and *Basidiomycota* (28.4%), with contributions from classes *Saccharomycetes* (30.8%), *Tremellomycetes* (18.5%), *Dothideomycetes* (21.6%) and *Sordariomycetes* (14.6%). However, there is no information about the dominant genus of fungi in previous studies [27,28]. *Purpureocillium* (4.5%), *Cladosporium* (2.5%) and *Pezicula* (2.4%) were the top three genera of fungi. The variations in proportion of the dominant species in previous studies on gut microbiota of GPs were probably caused by environmental or other host physiological and genetic factors [12].

Our study demonstrated the discrepancy between male and female GPs. We found that the male GPs harbor a higher abundance of phylum *Firmicutes* than female GPs with the contribution from genera *Streptococcus*. Meanwhile, the female GPs harbor a higher abundance of phylum *Proteobacteria* than male GPs with the contribution from genera *Escherichia/Shigella*. Some *Streptococcus* species are responsible for cases of endocarditis [29], erysipelas [30], and necrotizing fasciitis [31]. However, most of them are not pathogenic, and form part of the commensal microbiota of the intestine tract [32, 33]. The strains of *Escherichia/shigella* are also part of the normal flora of the gut, and can benefit their hosts by producing vitamin K2, and preventing colonization of the intestine with pathogenic bacteria, having a symbiotic relationship [34]. However, some virulent strains can cause gastrointestinal tract infections [35]. In recent years, the differences in gut microbiota between males and females had been successively reported [36]. The difference between males and females in the composition of mouse fecal flora has been examined by denaturing gradient gel electrophoresis (DGGE) [37]. However, the significant taxon cannot be detected by DGGE.. The gender effects on human gut bacteria were firstly observed for the genus *Prevotella*, which was affiliated to phylum *Bacteroides*, with higher levels in males than in females. Mueller et al suggested that the existence of a postpubescent gender bias in microbial diversity and representation of individual species became evident [38]. On the contrary to Mueller’s result, Haro et al found that the abundance of the *Bacteroides* was lower in men than women [39].

Gut bacteria has been reported to shift during gestation or pregnancy in human [19] and other mammals [40]. Koren et al demonstrated that pregnancy was associated with alterations to the gut microbiota based on animal (mice, mammal) model [41]. Ji et al discovered a tendency for the abundance of *Proteobacteria* to increase as pregnancy progressed in sow, even if all the sows share the same dominant phyla *Firmicutes*, *Proteobacteria* and *Bacteroidetes* of gut bacteria [16]. Meanwhile, Jost et al observed a significant decrease (*p* < 0.05) in *Lactobacillus*, suggesting that an underestimation of *Bacteroidetes* occurs during pregnancy [42]. In our study, we also found the shift from female to pregnant GPs that phylum *Firmicutes* underwent a significant increase (*p* <0.001) with the contribution of *Clostridium* and *Turicibacteria*. *Clostridium*, affiliated to *Firmicutes*, was known as a function of cellulose degradation [43]. However, some species of *Clostridium* like *Clostridium difficile* could be a threat to the health of pregnant host individuals, in particular, by causing diarrhea [44]. *Turicibacter* bacteria is commonly detected in the gastrointestinal tracts and feces of humans and animals, but their phylogeny, ecological role, and pathogenic potential remain unclear [45]. Han et al [46] reported that *Turicibacter belong to class Erysipelotrichia* which have been isolated from swine manure and increase in composition of the mouse gut microbiome for mice switched to diets high in fat [47]. There is evidence that pregnancy is a physiological state that is associated with shifts in gut microbiota [19]. Hence, the physiological and biochemical indexes of the pregnant GPs should be detected to be associated with the shifts in microbiota. If possible, a fecal transplant could be performed to improve the intestinal flora of non-pregnant giant pandas. Besides, the certain functions of the shift microbiota should be confirmed by using metagenomics.

In conclusion, we characterized the gut microbiota among the male, female and pregnant GPs. Through comparative analysis, we determined the discrepancy between male and female GPs which indicated that the male GPs harbor a higher abundance of phylum *Firmicutes* than female GPs with the contribution from genera *Streptococcus*. Meanwhile, the female GPs harbor a higher abundance of phylum *Proteobacteria* than male GPs with the contribution from genera *Escherichia/Shigella*. In addition, the shift in bacteria from female to pregnant GPs indicated that phylum *Firmicutes* increased significantly with the contribution from *Clostridium* in the gut, which may provide an opportunity to study possible associations with low reproduction of the GPs.

## References

[1] Peng Z, Zeng D, Wang Q, Niu L, Ni X, Zou F (2016). Decreased microbial diversity and *Lactobacillus* group in the intestine of geriatric giant pandas *Ailuropoda melanoleuca*. World J Microbiol Biotechnol.

[2] Wei F, Hu Y, Yan L, Nie Y, Wu Q, Zhang Z (2015). Giant pandas are not an evolutionary cul-de-sac: evidence from multidisciplinary research. Mol Biol Evol.

[3] Williams CL, Dill-McFarland KA, Vandewege MW, Sparks DL, Willard ST, Kouba AJ (2016). Dietary Shifts May Trigger Dysbiosis and Mucous Stools in Giant Pandas *Ailuropoda melanoleuca*. Front Microbiol.

[4] Li RQ, Fan W, Tian G, Zhu HM, He L, Cai J (2010). The sequence and de novo assembly of the giant panda genome. Nature.

[5] Delport TC, Power ML, Harcourt RG, Webster KN, Tetu SG (2016). Colony Location and Captivity Influence the Gut Microbial Community Composition of the Australian Sea Lion *Neophoca cinerea*. Appl Environ Microb.

[6] Clemente JC, Ursell LK, Parfrey LW, Knight R (2012). The impact of the gut microbiota on human health: an integrative view. Cell.

[7] Wei F, Wu Q, Hu Y, Huang G, Nie Y, Yan L (2018). Conservation metagenomics: a new branch of conservation biology. China Life sci.

[8] Ellis RJ, Bruce KD, Jenkins C, Stothard JR, Ajarova L, Mugisha L (2013). Comparison of the distal gut microbiota from people and animals in Africa. PloS one.

[9] Siddiqui H, Nederbragt AJ, Lagesen K, Jeansson SL, Jakobsen KS (2011). Assessing diversity of the female urine microbiota by high throughput sequencing of 16S rDNA amplicons. BMC microbiol.

[10] Huang C, Chen J, Wang J, Zhou H, Lu Y, Lou L (2017). Dysbiosis of Intestinal Microbiota and Decreased Antimicrobial Peptide Level in Paneth Cells during Hypertriglyceridemia-Related Acute Necrotizing Pancreatitis in Rats. Front Microbiol.

[11] Wang L, Wu D, Yan T, Wang L (2017). The impact of rumen cannulation on the microbial community of goat rumens as measured using 16S rRNA high-throughput sequencing. J Anim Physiol Anim Nutr (Berl).

[12] Li Y, Guo W, Han S, Kong F, Wang C, Li D (2015). The evolution of the gut microbiota in the giant and the red pandas. Sci Rep.

[13] Xue Z, Zhang W, Wang L, Hou R, Zhang M, Fei L (2015). The bamboo-eating giant panda harbors a carnivore-like gut microbiota, with excessive seasonal variations. mBio.

[14] Penders J, Thijs C, Vink C, Stelma FF, Snijders B, Kummeling I (2006). Factors influencing the composition of the intestinal microbiota in early infancy. Pediatrics.

[15] Kundu P, Blacher E, Elinav E, Pettersson S (2017). Our Gut Microbiome: The Evolving Inner Self. Cell.

[16] Ji Y, Kong X, Li H, Zhu Q, Guo Q, Yin Y (2017). Effects of dietary nutrient levels on microbial community composition and diversity in the ileal contents of pregnant Huanjiang mini-pigs. PloS one.

[17] Liao MJ, Zhu MY, Zhang ZH, Zhang AJ, Li GH, Sheng FJ (2003). Cloning and sequence analysis of FSH and LH in the giant panda *Ailuropoda melanoleuca*. Anim Reprod Sci.

[18] Wallace JG, Potts RH, Szamosi JC, Surette MG, Sloboda DM (2018). The murine female intestinal microbiota does not shift throughout the estrous cycle. PloS one.

[19] Gohir W, Whelan FJ, Surette MG, Moore C, Schertzer JD, Sloboda DM (2015). Pregnancy-related changes in the maternal gut microbiota are dependent upon the mother’s periconceptional diet. Gut microbes.

[20] Roulo RM, Fishburn JD, Amosu M, Etchison AR, Smith MA (2014). Dose response of Listeria monocytogenes invasion, fetal morbidity, and fetal mortality after oral challenge in pregnant and nonpregnant Mongolian gerbils. Infect Immun.

[21] Yang X, Cheng G, Li C, Yang J, Li J, Chen D (2017). The normal vaginal and uterine bacterial microbiome in giant pandas *Ailuropoda melanoleuca*. Microbiol Res.

[22] Magoc T, Salzberg SL (2011). FLASH: fast length adjustment of short reads to improve genome assemblies. Bioinformatics.

[23] Caporaso JG, Kuczynski J, Stombaugh J, Bittinger K, Bushman FD, Costello EK (2010). QIIME allows analysis of high-throughput community sequencing data. Nat methods.

[24] Edgar RC, Haas BJ, Clemente JC, Quince C, Knight R (2011). UCHIME improves sensitivity and speed of chimera detection. Bioinformatics.

[25] Li Y, Hu X, Yang S, Zhou J, Zhang T, Qi L (2017). Comparative Analysis of the Gut Microbiota Composition between Captive and Wild Forest Musk Deer. Front Microbiol.

[26] Jiang XT, Peng X, Deng GH, Sheng HF, Wang Y, Zhou HW (2013). Illumina Sequencing of 16S rRNA Tag Revealed Spatial Variations of Bacterial Communities in a Mangrove Wetland. Microb ecol.

[27] Ai S, Zhong Z, Peng G, Wang C, Luo Y, He T (2014). Intestinal fungal diversity of sub-adult giant panda. Wei sheng wu xue bao.

[28] Tun HM, Mauroo NF, Yuen CS, Ho JCW, Wong MT, Leung FCC (2014). Microbial Diversity and Evidence of Novel Homoacetogens in the Gut of Both Geriatric and Adult Giant Pandas *Ailuropoda melanoleuca*. PloS one.

[29] Mettananda S, Kamalanathan P, Dhananja Namalie K (2018). *Streptococcus bovis* - unusual etiology of meningitis in a neonate with Down syndrome: a case report. J Med Case Rep.

[30] Piedimonte S, Almohammadi M, Lee TC (2018). Group B *Streptococcus* tricuspid valve endocarditis with subsequent septic embolization to the pulmonary artery: A case report following elective abortion. Obstet Med.

[31] Deneubourg DL, Catherine Z, Lejuste P, Breton P (2018). Periorbital Necrotizing Fasciitis Induced by *Streptococcus* pyogenes: A Case Report and Clarification. J Oral Maxillofac Surg.

[32] Couvigny B, de Wouters T, Kaci G, Jacouton E, Delorme C, Dore J (2015). Commensal *Streptococcus salivarius* Modulates PPARgamma Transcriptional Activity in Human Intestinal Epithelial Cells. PloS one.

[33] Kambarev S, Pecorari F, Corvec S (2018). Novel Tn916-like elements confer aminoglycoside/macrolide co-resistance in clinical isolates of *Streptococcus gallolyticus* ssp. gallolyticus. J Antimicrob Chemother.

[34] Karl JP, Fu X, Wang X, Zhao Y, Shen J, Zhang C (2015). Fecal menaquinone profiles of overweight adults are associated with gut microbiota composition during a gut microbiota-targeted dietary intervention. Am J Clin Nutr.

[35] Siegman-Igra Y, Azmon Y, Schwartz D (2012). Milleri group streptococcus--a stepchild in the viridans family. Eur J Clin Microbiol Infect Dis.

[36] Yurkovetskiy L, Burrows M, Khan AA, Graham L, Volchkov P, Becker L (2013). Gender Bias in Autoimmunity Is Influenced by Microbiota. Immunity.

[37] Fushuku S, Fukuda K (2008). Gender difference in the composition of fecal flora in laboratory mice, as detected by denaturing gradient gel electrophoresis (DGGE). Exp Anim.

[38] Mueller S, Saunier K, Hanisch C, Norin E, Alm L, Midtvedt T (2006). Differences in fecal microbiota in different European study populations in relation to age, gender, and country: a cross-sectional study. Appl Environ Microbiol.

[39] Haro C, Rangel-Zúñiga OA, Alcalá-Díaz JF, Gómez-Delgado F, Pérez-Martínez P, Delgado-Lista J (2016). Intestinal Microbiota Is Influenced by Gender and Body Mass Index. PloS one.

[40] Ji Y, Guo Q, Yin Y, Blachier F, Kong X (2018). Dietary proline supplementation alters colonic luminal microbiota and bacterial metabolite composition between days 45 and 70 of pregnancy in Huanjiang mini-pigs. J Anim Sci Biotechnol.

[41] Koren O, Goodrich JK, Cullender TC, Spor A, Laitinen K, Backhed HK (2012). Host remodeling of the gut microbiome and metabolic changes during pregnancy. Cell.

[42] Jost T, Lacroix C, Braegger C, Chassard C (2014). Stability of the maternal gut microbiota during late pregnancy and early lactation. Curr microbiol.

[43] Wei FW, Hu YB, Zhu LF, Bruford MW, Zhan XJ, Zhang L (2012). Black and white and read all over: the past, present and future of giant panda genetics. Mol Ecol.

[44] Nada AM, Mohsen RA, Hassan YM, Sabry A, Soliman NS (2018). Does Saline Enema During the First Stage of Labour Reduce the Incidence of *Clostridium difficile* Colonization in Neonates? Randomized Controlled Trial. J Hosp Infect.

[45] Tao S, Tian P, Luo Y, Tian J, Hua C, Geng Y (2017). Microbiome-Metabolome Responses to a High-Grain Diet Associated with the Hind-Gut Health of Goats. Front Microbiol.

[46] Han I, Congeevaram S, Ki DW, Oh BT, Park J (2011). Bacterial community analysis of swine manure treated with autothermal thermophilic aerobic digestion. Appl microbiol biotechnol.

[47] Greiner T, Backhed F (2011). Effects of the gut microbiota on obesity and glucose homeostasis. TEM.

