# Supplementary material for: Comparative Analysis of Gut Microbiota Among the Male, Female and Pregnant Giant Pandas (Ailuropoda Melanoleuca)
Source: Open Life Sci. 2019 Jul 23;14:288–98. doi: 10.1515/biol-2019-0032 (PMC7874769; doi:10.1515/biol-2019-0032)
Supplement: Supplementary file 1 [file biol-14-288_sm.pdf]

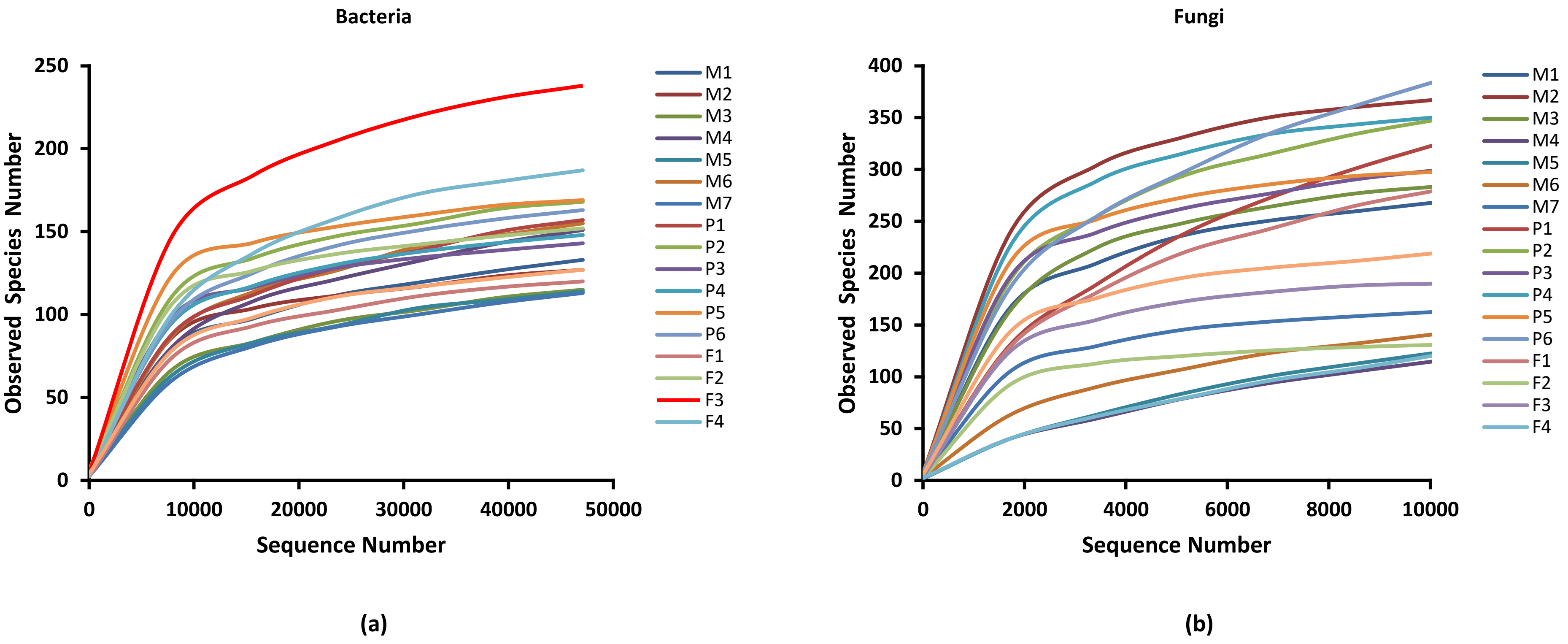

Supplementary Figure 1. Rarefaction curves. The former reflects the rationality of our sequencing data size and abundance of species in feces samples indirectly

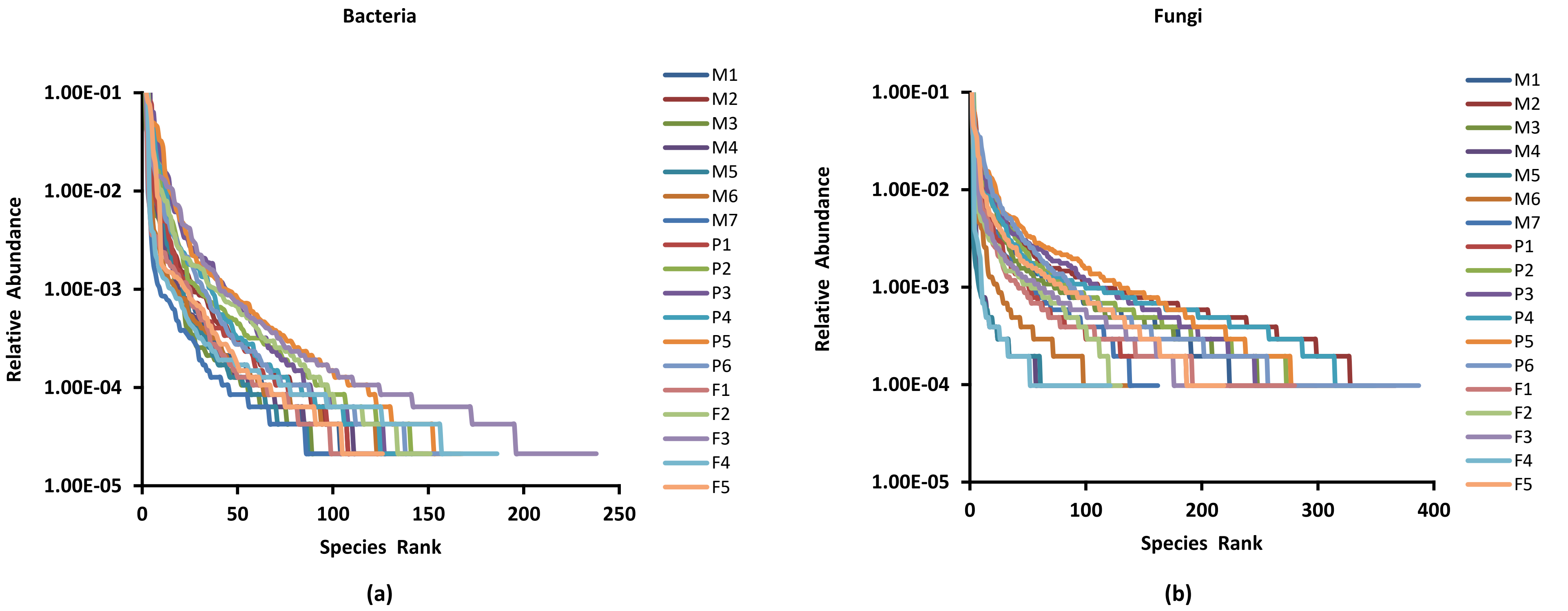

Supplementary Figure 2. Rank abundance curves. Wider span of curves reveals higher relative abundance of species in horizontal direction and the smoothness of curves showed the evenness of bacterial and fungal species in samples vertically

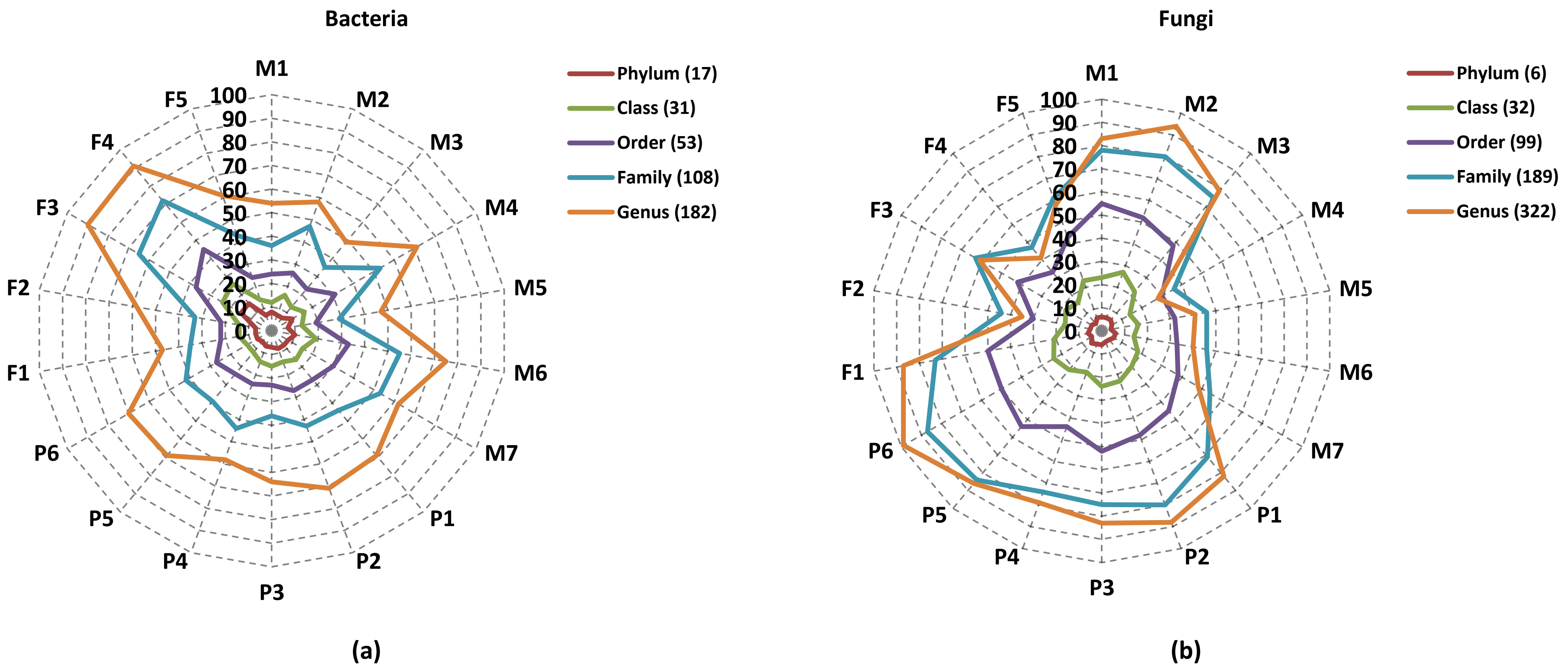

Supplementary Figure 3. Overview of the classification, the numbers in brackets stand for the total amount at each level of all samples

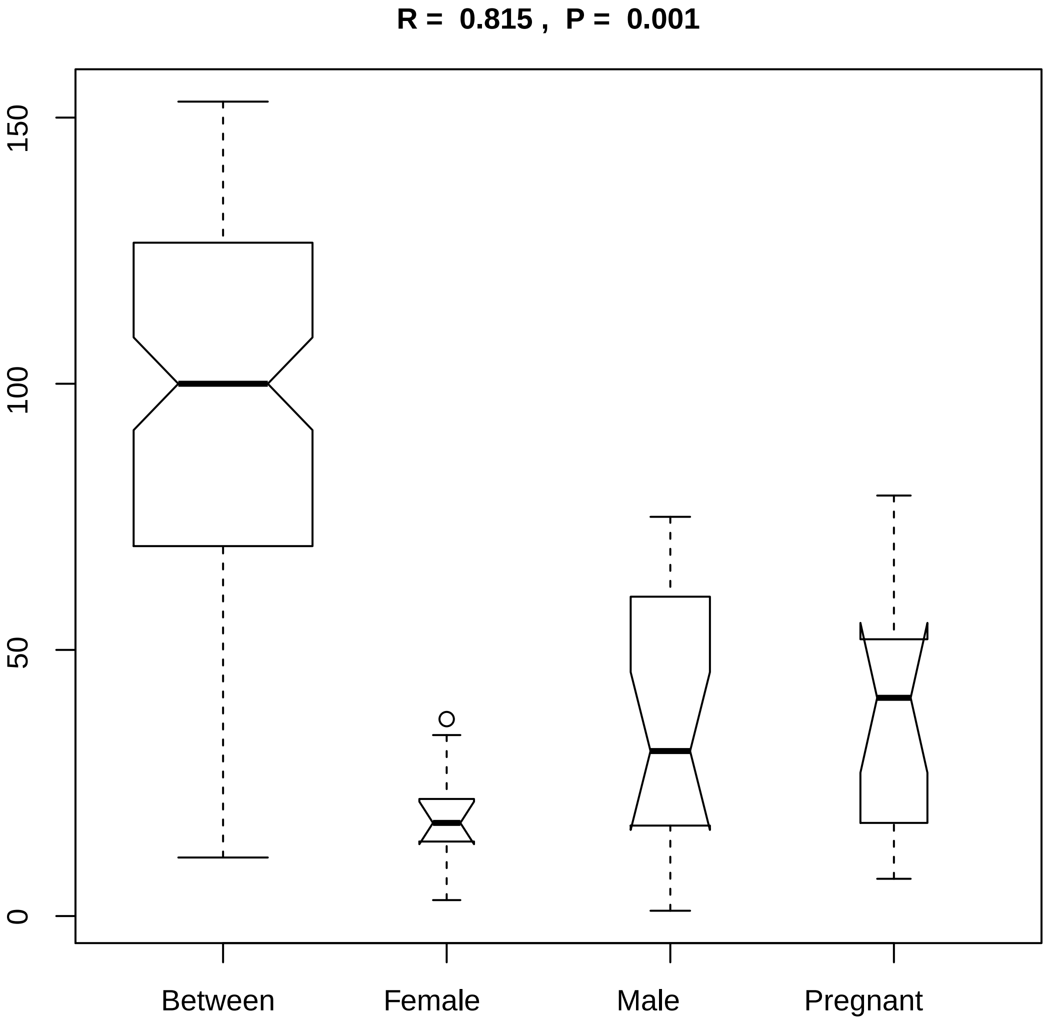

Supplementary Figure 4 ANOSIM for Pcoa analysis in 16S high throughput sequencing

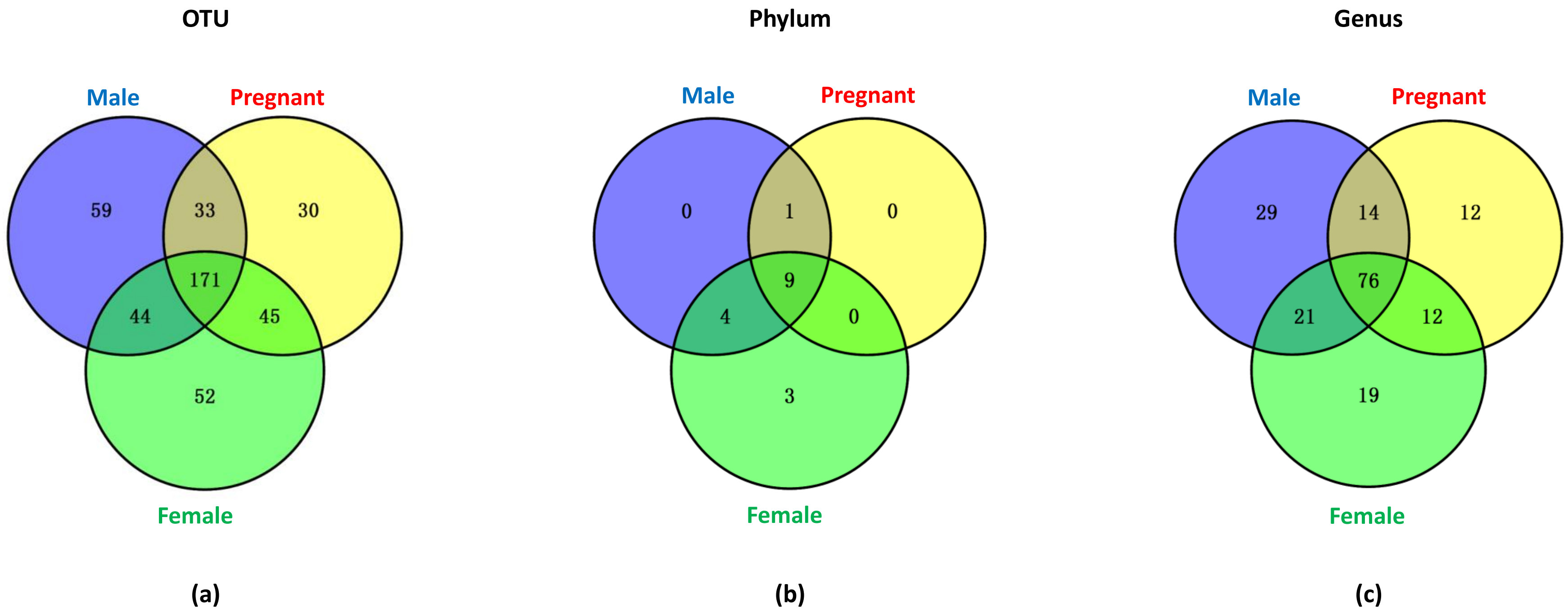

Supplementary Figure 5. Venn diagrams of the OTUs, phyla and genera among the male, female and pregnant groups

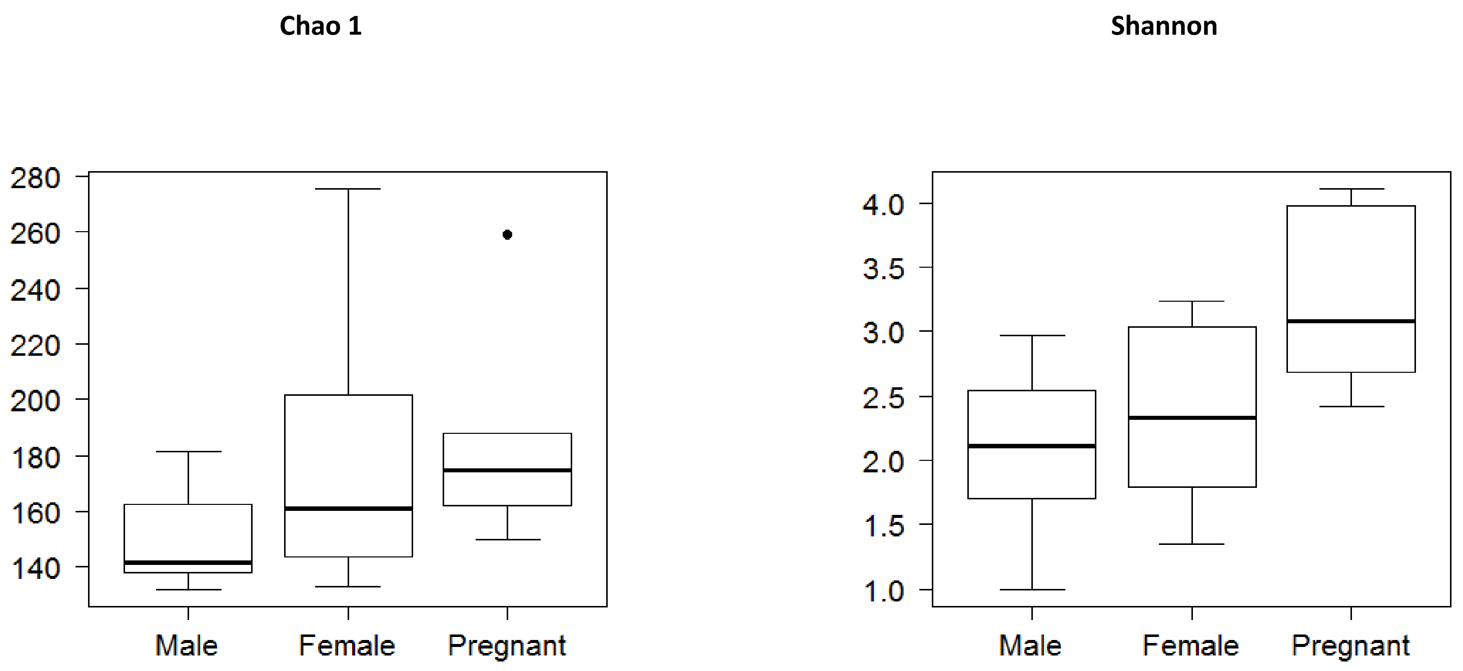

Supplementary Figure 6 Boxplot of Chao1 and Shannon index among the male, female and pregnant groups

Supplementary Table 1 Alpha diversity of bacteria in feces samples from male, female and pregnant giant pandas

Sample name	observed species	Shannon	Simpson	Chao1	ACE	Goods coverage
M1	133	2.497	0.674	152.773	159.369	0.999
M2	127	2.974	0.795	141.615	140.313	1.000
M3	115	1.399	0.339	140.071	138.146	0.999
M4	151	2.574	0.770	181.370	190.344	0.999
M5	114	2.106	0.598	136.235	138.939	0.999
M6	155	1.999	0.611	172.600	180.400	0.999
M7	113	0.994	0.302	131.900	134.393	0.999
F1	120	1.346	0.372	132.833	134.436	1.000
F2	152	3.040	0.748	161.000	165.504	1.000
F3	238	3.233	0.695	275.625	266.067	0.999
F4	187	1.790	0.589	201.531	204.540	0.999
F5	127	2.333	0.629	143.867	141.774	1.000
P1	157	2.685	0.732	259.083	214.164	0.999
P2	168	3.120	0.768	187.895	187.574	0.999
P3	143	3.978	0.890	149.800	154.455	1.000
P4	148	3.039	0.721	161.800	164.334	0.999
P5	169	4.110	0.896	174.913	178.985	1.000
P6	163	2.421	0.585	175.037	178.675	0.999

Supplementary Table 2 Alpha diversity of fungi in feces samples from male, female and pregnant giant pandas

Sample name	Observed species	Shannon	Simpson	chao1	ACE	goods coverage
M1	269	4.128	0.774	299.441	292.988	0.996
M2	368	6.044	0.955	395.333	384.342	0.996
M3	284	4.443	0.836	300.650	301.448	0.996
M4	116	1.581	0.567	189.750	213.562	0.994
M5	124	1.361	0.517	196.000	230.211	0.994
M6	142	1.641	0.364	178.667	183.071	0.996
M7	163	3.471	0.768	184.667	175.860	0.997
F1	281	3.151	0.694	361.100	363.465	0.991
F2	131	2.214	0.441	138.333	135.770	0.999
F3	190	2.880	0.561	196.562	195.171	0.999
F4	122	1.521	0.500	246.250	244.577	0.993
F5	220	4.145	0.790	243.375	239.806	0.997
P1	326	3.769	0.792	493.759	509.022	0.986
P2	349	5.263	0.918	409.958	401.940	0.992
P3	300	5.412	0.910	361.875	336.231	0.995
P4	351	5.106	0.855	373.966	365.001	0.996
P5	298	6.270	0.970	303.775	307.926	0.998
P6	387	5.671	0.949	539.054	533.679	0.987
